# A Novel AlGaN/GaN Transient Voltage Suppression Diode with Bidirectional Clamp Capability

**DOI:** 10.3390/mi13020299

**Published:** 2022-02-14

**Authors:** Zhiyuan He, Yijun Shi, Yun Huang, Yiqiang Chen, Hongyue Wang, Lei Wang, Guoguang Lu, Yajie Xin

**Affiliations:** The Science and Technology on Reliability Physics and Application of Electronic Component Laboratory, China Electronic, Product Reliability and Environmental Testing Research Institute, Guangzhou 510610, China; hezhiyuan1988@126.com (Z.H.); huangyun@ceprei.com (Y.H.); wanghongyue@pku.edu.cn (H.W.); leiwang@ceprei.com (L.W.); luguog@126.com (G.L.); 201811022423@std.uestc.edu.cn (Y.X.)

**Keywords:** transient voltage suppression, TVS diode, GaN HEMT, Transmission Line Pulsing

## Abstract

This work proposes a novel AlGaN/GaN transient voltage suppression (TVS) diode (B-TVS-D) with bidirectional clamp capability, which consists of a small-size AlGaN/GaN monolithic bidirectional switch, two 2DEG-based current-limiting resistors (*R*_1A_/*R*_1C_, in parallel connection between the gate electrodes and the neighboring ohmic-contact electrodes (anode/cathode)), and a 2DEG-based proportional amplification resistor (*R*_2_, in parallel connection between two gate electrodes). It is demonstrated that the proposed B-TVS-D possesses a symmetrical triggering voltage (*V*_trig_) and a high secondary breakdown current (*I*_s_, over 8 A, corresponding to 12 kV human body model failure voltage) in different directional electrostatic discharge (ESD) events. The proposed diode can effectively enhance the electrostatic discharge robustness for the GaN-based power system. It is also verified that *R*_1A_/*R*_1C_ and *R*_2_ have an important impact on *V*_trig_ of the proposed B-TVS-D. Both the decrease in *R*_2_ and increase in *R*_1A_/*R*_1C_ can lead to the decrease of *V*_trig_. In addition, the proposed B-TVS-D can be fabricated on the conventional p-GaN HEMT platform, making the ESD design of the GaN-based power system more convenient.

## 1. Introduction

Gallium nitride (GaN) has several notable material properties (such as high electron saturation velocity, high electron mobility, and wide bandgap), which makes GaN-based diodes and high electron mobility transistors (HEMTs) attract broad attention in power electronics [1,2,3,4,5,6]. However, the possibility of an electrostatic discharge (ESD) event always threatens the reliability of GaN-based HEMTs and diodes [7,8,9,10,11,12,13,14,15]. In our previous study, it is found that the human body model failure voltage (*V*_HBM_) of the p-gate structure of some commercial HEMTs is less than 0.5 kV, which is far below the trade standard (2 kV) [16,17]. Some researchers have studied and analyzed the ESD protection ability of a GaN-based Schottky barrier diode [11,12,13,14,15]. Although GaN-based Schottky barrier diodes can discharge the positive electrostatic charge, the forward triggering voltage of GaN-based Schottky barrier diodes is less than 2 V, which is lower than the working voltage (+6 V) of p-GaN HEMT. In addition, GaN-based Schottky barrier diodes can discharge forward electrostatic charge but cannot discharge reverse electrostatic charge. Thus, GaN-based Schottky barrier diodes cannot effectively protect the gate structure of commercial p-GaN HEMT from ESD damage. To effectively protect the p-GaN HEMTs from ESD damage, it is necessary to ameliorate the ESD robustness for the p-gate structure. In this connection, Yajie Xin has proposed a unidirectional AlGaN/GaN transient voltage suppression (TVS) diode [18], which can be self-triggered by a desirable value in the unidirectional transient ESD event. However, in some fields, namely AC circuit and communication systems, a TVS diode with the capability of bidirectional transient voltage suppression is needed [19]. To achieve this goal, we have proposed a bidirectional AlGaN/GaN TVS diode, which possesses a symmetrical triggering voltage (*V*_trig_) and a high secondary breakdown current (*I*_s_) in different directional electrostatic discharge events [19]. However, to obtain a required triggering voltage, a relatively large capacitor is needed, which will also obviously decrease the *I*_s_ of the bidirectional AlGaN/GaN TVS diode; i.e., the protection capability of that bidirectional AlGaN/GaN TVS diode will be obviously weakened, as described in our previous work. Therefore, there still is an urgent need for optimizing or redesigning the bidirectional AlGaN/GaN TVS diode.

In this work, we have proposed a novel bidirectional AlGaN/GaN TVS diode (B-TVS-D), which consists of a small-size AlGaN/GaN monolithic bidirectional switch (MBS), two current-limiting resistors (*R*_1A_/*R*_1C_, in parallel connection between the gate electrodes and the neighboring ohmic-contact electrodes (anode/cathode)), and a 2DEG-based proportional amplification resistor (*R*_2_, in parallel connection between two gate electrodes). The proposed B-TVS-D possesses a symmetrical *V*_trig_ and a high *I*_s_ in different directional electrostatic discharge events. This work is organized as follows. First, the structures and mechanisms of the unidirectional TVS diode and the proposed B-TVS-D are presented. Then, the characteristics of the proposed B-TVS-D, and the influence of *R*_1C_/*R*_1A_ and *R*_2_ are investigated. Finally, the conclusions are drawn.

## 2. Materials and Methods

**Figure 1:**
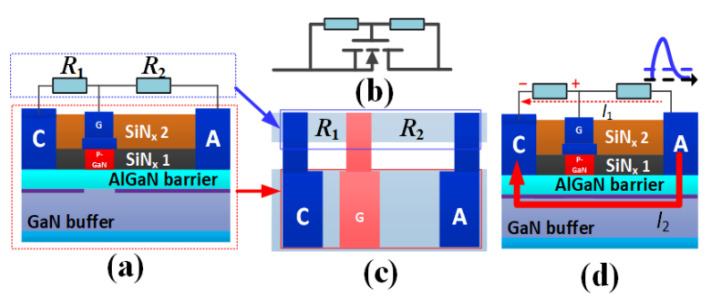
(**a**) The structure, (**b**) equivalent circuit, (**c**) plane layout and (**d**) working mechanism of the unidirectional TVS diode [18].

Before introducing the proposed AlGaN/GaN B-TVS-D, it is necessary to introduce the unidirectional AlGaN/GaN TVS diode developed in previous work [18]. Figure 1a, b give the schematic structure of the unidirectional TVS diode, containing a p-GaN HEMT, a 2DEG-based current-limiting resistor (*R*_1_) and a 2DEG-based proportional amplification resistor (*R*_2_). *R*_1_ is parallelly connected between the diode’s gate electrode and the cathode electrode, and *R*_2_ is parallelly connected between the diode’s gate electrode and the anode electrode. It is obvious that the unidirectional TVS diode can be fabricated on the conventional p-GaN HEMT platform.

**Figure 2:**
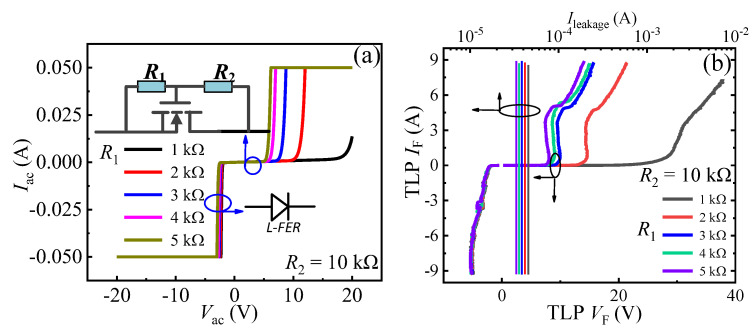
The bidirectional static leakage (**a**) and TLP *I-V* characteristics (**b**) of the unidirectional TVS diode with *R*_2_ = 10 kΩ and different *R*_1_.

The mechanism of the unidirectional TVS diode is exhibited in Figure 1d. During the forward transient ESD event (from A to C), a small amount transient electrostatic charges will flow through *R*_1_ and *R*_2_. Then, there is a transient voltage drop between the diode’s gate and the cathode. When the transient voltage drop is larger than the threshold voltage (*V*_th_) of the p-GaN HEMT, the unidirectional TVS diode will be triggered. Then, the forward transient electrostatic charges can be discharged through the unidirectional TVS diode (Figure 1d). Thereby, the ESD damage can be avoided. As can be surmised, *R*_1_ and *R*_2_ play an important role on the forward triggering voltages (*V*_trig_F_) of the unidirectional TVS diode. The increase in *R*_1_ or decrease in *R*_2_ can lead to the decrease in *V*_trig_F_ (Figure 2) [18]. So, through changing *R*_1_ or *R*_2_, a desirable *V*_trig_F_ can also be obtained. However, in the reverse ESD event (from C to A), the unidirectional TVS diode is triggered by a very low voltage, just like a lateral field effect rectifier (L-FER). And the changes in *R*_1_ and *R*_2_ have nearly no effect on the device’s reverse triggering voltages (*V*_trig_R_). So, the unidirectional TVS diode is incapable of clamping the potential to be a desirable voltage in the reverse ESD event. To realize the target of bidirectional clamp, a bidirectional AlGaN/GaN TVS diode is proposed in this work.

**Figure 3:**
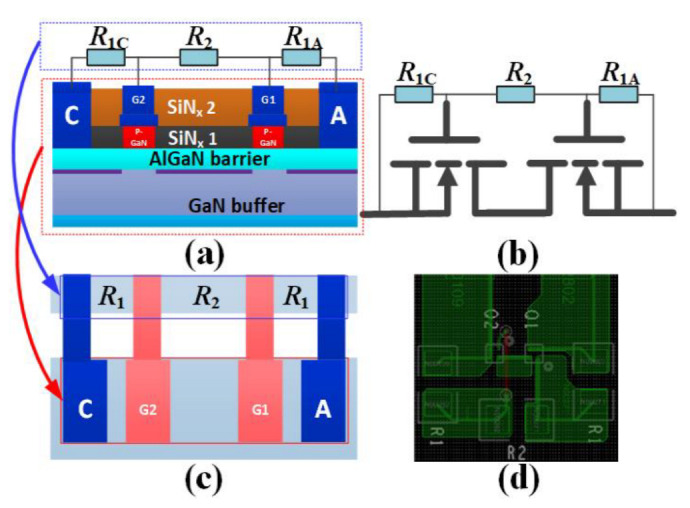
(**a**) The structure, (**b**) equivalent circuit and (**c**) plane layout of the proposed B-TVS-D. (**d**) The equivalent structure configured by the chip resistor and the p-GaN HEMT.

Figure 3a,b give the structure of the proposed B-TVS-D. The diode consists of a small-size AlGaN/GaN MBS, two 2DEG-based current-limiting resistors (*R*_1C_/*R*_1A_), and a 2DEG-based proportional amplification resistor (*R*_2_). *R*_1C_/*R*_1A_ is in parallel connection between the gate electrodes and the neighboring ohmic-contact electrodes (anode/cathode), and *R*_2_ is connected in parallel between two gate electrodes. It can also be found that the proposed B-TVS-D can be fabricated on the traditional p-GaN HEMT platform. Moreover, the required 2DEG-based resistors can be easily integrated through changing the length of the 2DEG-based resistors. For example, when the width of the 2DEG-based resistor is 3 μm, the required length of the 2DEG-based resistor is less than 100 μm, and the corresponding area is less than 0.0003 mm^2^, which makes up no more than 0.01% of the conventional HEMT’s area [20].

**Figure 4:**
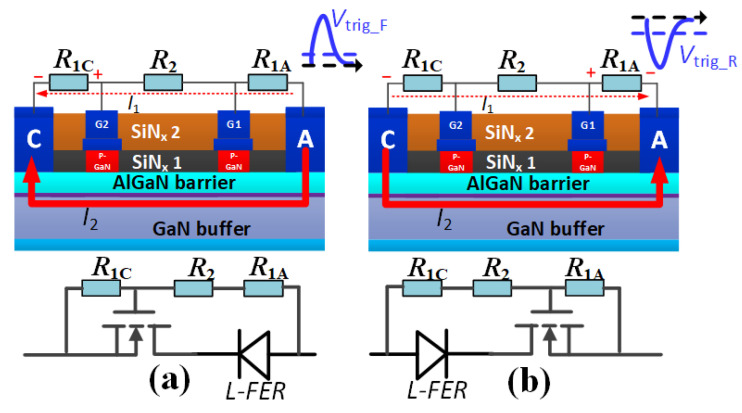
Working mechanism of the proposed AlGaN/GaN B-TVS-D: (**a**) During the forward ESD event, (**b**) During the reverse ESD event.

The mechanism of the proposed B-TVS-D is exhibited in Figure 4. In both the forward and reverse ESD event, the proposed B-TVS-D is similar to the combination of a unidirectional TVS diode and a L-FER, but in different directions. During the forward ESD event, the diode’s first gate structure and anode act as the L-FER, and the diode’s second gate structure, cathode, *R*_1C_, *R*_1A_ and *R*_2_ act as the unidirectional AlGaN/GaN TVS diode (Figure 4a). The L-FER can be turned on at a very low voltage, as shown in Figure 2. The transient electrostatic charges will arouse a forward current flowing through *R*_1C_, *R*_1A_ and *R*_2_, and lead to a transient voltage drop between the diode’s second gate electrode and cathode electrode. When the transient voltage drop is larger than *V*_th_ of the second gate structure of the AlGaN/GaN MBS, the MBS will be turned on. Then, the forward transient electrostatic charge can be released through the proposed B-TVS-D. Through changing *R*_1C_, *R*_1A_ and *R*_2_, the diode can be triggered by a desirable value in the forward ESD event, just as for the unidirectional TVS diode. Similarly, the proposed B-TVS-D can also be triggered by a desirable value in the reverse ESD event. During the reverse ESD event, the diode’s second gate structure and cathode act as the L-FER, and the diode’s first gate structure, anode, *R*_1C_, *R*_1A_ and *R*_2_ act as the unidirectional TVS diode. The transient voltage induced by the electrostatic charges will arouse a reverse current flowing through *R*_1C_, *R*_1A_ and *R*_2_, and lead to a transient voltage drop between the diode’s first gate electrode and the anode electrode. When the voltage drop is larger than *V*_th_ of the first gate structure of the AlGaN/GaN MBS, it will be turned on. Then, the reverse transient electrostatic charge can discharge through the proposed B-TVS-D.

To reduce the validation cost, the working mechanism of the proposed B-TVS-D is verified by the equivalent structure configured by the commercial p-GaN HEMT (EPC2036) [21] and the chip resistor, as shown in Figure 3d. The areas of EPC2036 and the 2DEG-based resistors are only about 0.81 mm^2^ and 0.0003 mm^2^, respectively. So, the area of the proposed B-TVS-D is about 1.6203 mm^2^, which makes up no more than 5% of the conventional HEMT’s area [20]. In this work, the proposed B-TVS-D is tested by our self-developed transmission line pulsing (TLP) measurement system. The rising time and pulse width are set to be 2 ns and 100 ns, respectively. Since the practical *I*_s_ of the proposed B-TVS-D cannot be obtained for the limitation of our self-developed TLP measurement system, *I*_s_ in this work is defined at the transient applied voltage reaching to system’s limit (1000 V). *V*_trig_ of the proposed B-TVS-D is defined at the transient TLP current of 0.1 A.

## 3. Results and Discussion

**Figure 5:**
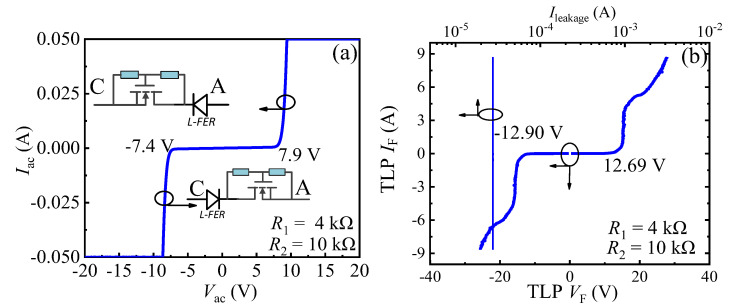
The bidirectional static leakage (**a**) and TLP I-V characteristic (**b**) of the proposed B-TVS-D, with *R*_1C_/*R*_1A_ = 4 kΩ and *R*_2_ = 10 kΩ.

Figure 5 plots the bidirectional static leakage and TLP *I-V* characteristic for the proposed B-TVS-D with *R*_1C_/*R*_1A_ = 4 kΩ and *R*_2_ = 10 kΩ. As stated above, in both the forward and reverse transient ESD event, the proposed B-TVS-D is similar to the combination of the unidirectional TVS diode and L-FER, but in different directions. Thereby, the proposed diode can possess a symmetrical bidirectional static leakage current characteristic and a symmetrical bidirectional TLP *I-V* characteristic, which are different from those of the unidirectional TVS diode. For the proposed B-TVS-D with *R*_1C_/*R*_1A_ = 4 kΩ and *R*_2_ = 10 kΩ, the diode’s forward turn-on voltage of the static leakage current (*V*_T_F_, defined at the static leakage current of 1 mA) is 7.9V, which is close to its reverse turn-on voltage (*V*_T_R_ = −7.4 V, defined at the static leakage current of −1 mA). For the unidirectional AlGaN/GaN TVS diode with *R*_1_ = 4 kΩ and *R*_2_ = 10 kΩ, the diode’s *V*_T_F_ is about 5 V, and the value is different from its *V*_T_R_ (~−2 V). Thereby, the proposed AlGaN/GaN B-TVS-D with *R*_1C_/*R*_1A_ = 4 kΩ and *R*_2_ = 10 kΩ will not obviously increase the leakage for GaN-based power system with the static applied voltage in the range from −7.4 V to 7.9 V. Through changing *R*_1C_, *R*_1A_ and *R*_2_, a desirable turn-on voltage (*V*_T_F_ and *V*_T_R_) can be acquired for the proposed B-TVS-D, which will be described in the following part.

In the positive TLP test, it is found that the proposed B-TVS-D with *R*_1C_/*R*_1A_ = 4 kΩ and *R*_2_ = 10 kΩ can be triggered by a low voltage of 12.69 V; the value is close to its *V*_trig_R_ (=−12.9 V). Thus, in different directional transient electrostatic discharge events, the proposed diode can effectively clamp the potential to a low value. Through changing *R*_1C_, *R*_1A_ and *R*_2_, a desirable triggering voltage (*V*_trig_F_ and *V*_trig_R_) can be acquired for the proposed B-TVS-D, which is different from the unidirectional TVS diode and will be described in the following part. Besides, in both the different directional TLP tests, the proposed B-TVS-D possesses a high *I*_S_ of over 8 A, showing that the proposed diode can usefully discharge the transient electrostatic charges in different directional transient electrostatic discharge events. Thus, the proposed diode can effectively enhance the electrostatic discharge robustness for the GaN-based power system.

**Figure 6:**
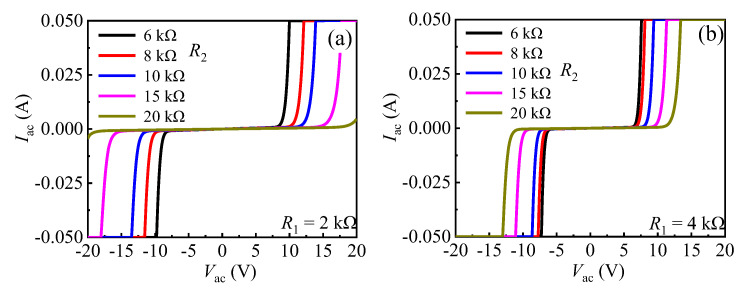
The bidirectional static leakage of the proposed B-TVS-D with different *R*_2_: (**a**) *R*_1C_/*R*_1A_ = 2 kΩ; (**b**) *R*_1C_/*R*_1A_ = 4 kΩ.

**Figure 7:**
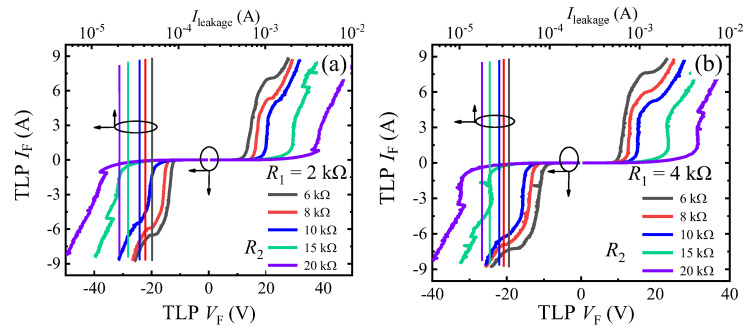
The bidirectional TLP *I-V* characteristics of the proposed B-TVS-D with different *R*_2_: (**a**) *R*_1C_/*R*_1A_ = 2 kΩ; (**b**) *R*_1C_/*R*_1A_ = 4 kΩ.

It can be easily speculated that *R*_1C_/*R*_1A_ and *R*_2_ pay an important role on the bidirectional static leakage and TLP *I-V* characteristics of the proposed B-TVS-D. Therefore, the characteristics of the proposed B-TVS-D with different *R*_1C_/*R*_1A_ and *R*_2_ are investigated here. First, the bidirectional static leakage and TLP *I-V* characteristics of the proposed B-TVS-D with different *R*_2_ are presented in Figure 6 and Figure 7. From Figure 6, it can be seen that the turn-on voltage of the static bidirectional leakage current (*V*_T_) is increased with the increase in *R*_2_. With *R*_2_ increased from 6 kΩ to 20 kΩ, *V*_T_ are increased from 6.4 V to 11.2 V for the proposed AlGaN/GaN B-TVS-D with *R*_1C_/*R*_1A_ = 4 kΩ, and increased from 8.2 V to 18.5 V for the diode with *R*_1C_/*R*_1A_ = 2 kΩ. This is because the increase in *R*_2_ will decrease the voltage drop at *R*_1C_ and *R*_1A_, subsequently reducing the voltage at the second gate structure in the forward conduction state or reducing the voltage at the first gate structure in the reverse conduction state. Therefore, a higher applied voltage is needed to turn on the normally-off gate structure of the AlGaN/GaN MBS in the proposed B-TVS-D. Hence, through changing *R*_2_, a desirable *V*_T_ can be acquired for the proposed B-TVS-D.

**Figure 8:**
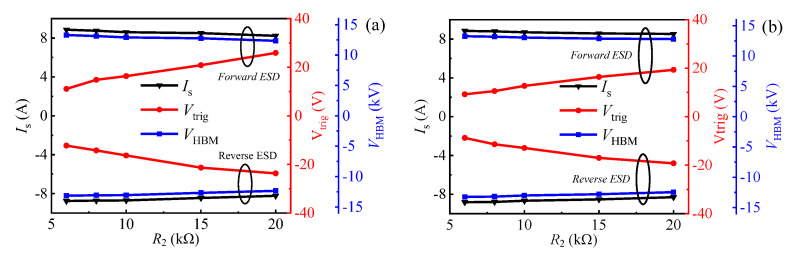
*I*_S_, *V*_trig_ and *V*_HBM_ of the proposed B-TVS-D with different *R*_2_: (**a**) *R*_1C_/*R*_1A_ = 2 kΩ; (**b**) *R*_1C_/*R*_1A_ = 4 kΩ.

Similarly, in the transient ESD event, the forward and reverse triggering voltages (*V*_trig_F_ and *V*_trig_R_) of the proposed B-TVS-D are also increased with the increase in *R*_2_. With *R*_2_ increased from 6 kΩ to 20 kΩ, the triggering voltages are increased from 9.2 V to 19.0 V for the proposed B-TVS-D with *R*_1C_/*R*_1A_ = 4 kΩ, and increased from 11 V to 25.9 V for the proposed B-TVS-D with *R*_1C_/*R*_1A_ = 2 kΩ. So, through changing *R*_2_, a desirable triggering voltage can also be acquired for the proposed B-TVS-D (Figure 8). It should be noted that the proposed B-TVS-D with low triggering voltage will possess a low turn-on voltage of the static leakage current. The designers should try to avoid premature turn-on of the static leakage current before obtaining low triggering voltage in the transient ESD event. All the proposed B-TVS-D possess a high *I*_S_ over than 8 A. Correspondingly, the equivalent HBM failure voltage (*V*_HBM_ = *I*_S_ × 1500 Ω) of the proposed B-TVS-D reaches to 12 kV; the value is higher than that of the bidirectional TVS diode in our previous work [19]. For the bidirectional AlGaN/GaN TVS diode in our previous work, both its *V*_trig_ and *I*_S_ are dependent on its capacitor. With its capacitor increasing from 5 pF to 25 pF, its *V*_trig_ is decreased from 18 V to 7 V, but its *I*_S_ is also decreased from 7 A to 3 A. Thus, to obtain a required triggering voltage, the diode’s protection capability will be weakened. To increase *I*_S_ for that bidirectional AlGaN/GaN TVS diode, a lager chip size is needed, which will increase corresponding costs. However, the relatively low static leakage current of the bidirectional AlGaN/GaN TVS diode may attract interest in some application.

**Figure 9:**
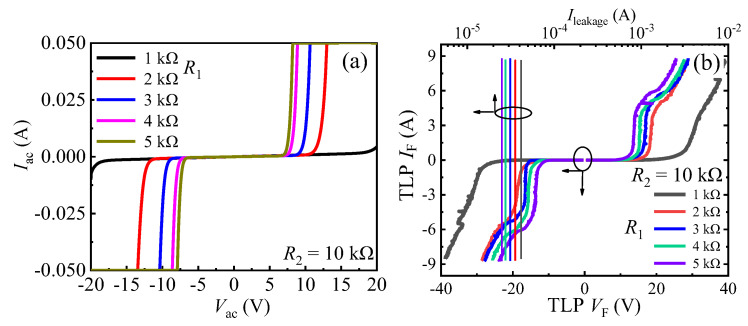
The bidirectional static leakage (**a**) and TLP *I-V* characteristics (**b**) of the proposed B-TVS-D with different *R*_1C_/*R*_1A_. *R*_2_ =10 kΩ.

**Figure 10:**
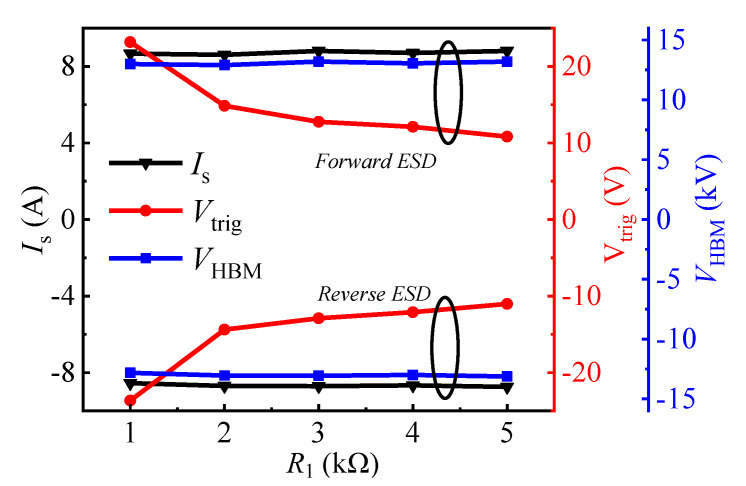
*I*_S_, *V*_trig_ and *V*_HBM_ of the proposed B-TVS-D with different *R*_1C_/*R*_1A_. *R*_2_ = 10 kΩ.

The influence of *R*_1C_/*R*_1A_ on the bidirectional static leakage and TLP *I-V* characteristics of the proposed B-TVS-D is presented in Figure 9 and Figure 10. The diode’s *V*_T_ is decreased with the increase in *R*_1C_/*R*_1A_. With *R*_1C_/*R*_1A_ increased from 1 kΩ to 5 kΩ, *V*_T_ is decreased from 18.2 V to 6.9 V for the proposed B-TVS-D with *R*_2_ = 10 kΩ. This is because the increase in *R*_1_ will increase the voltage drop at *R*_1C_ and *R*_1A_, subsequently increasing the voltage at the second gate structure in the forward conduction state or increasing the voltage at the first gate structure in the reverse conduction state. Therefore, a lower applied voltage is needed to turn on the normally-off gate structure of the AlGaN/GaN MBS in the proposed B-TVS-D. Through changing *R*_1_, a desirable *V*_T_ can also be acquired for the proposed B-TVS-D. Similarly, in the ESD event, the triggering voltages of the proposed B-TVS-D are also decreased with the increase in *R*_1_. With *R*_1C_/*R*_1A_ increased from 1 kΩ to 5 kΩ, the triggering voltages are increased from 23.2 V to 10.8 V for the proposed B-TVS-D with *R*_2_ = 10 kΩ. Through changing *R*_1_, a desirable triggering voltage can also be acquired for the proposed B-TVS-D (Figure 10). It should be noted that although increasing *R*_1C_/*R*_1A_ will lead to a low triggering voltage, it will also reduce response speed of the proposed B-TVS-D, as stated in our previous work [18].

## 4. Conclusions

In summary, a bidirectional AlGaN/GaN TVS diode is proposed and investigated. The proposed B-TVS-D features a small-size AlGaN/GaN monolithic bidirectional switch, two current-limiting resistors in parallel connection between the gate electrodes and the neighboring ohmic-contact electrodes (anode/cathode), and a proportional amplification resistor in parallel connection between two gate electrodes. It is demonstrated that the proposed B-TVS-D can be triggered by a desirable voltage and possesses an *I*_S_ over 8 A (corresponding to 12 kV *V*_HBM_) in different directional transient electrostatic discharge events. Further, it is also verified that *R*_1A_/*R*_1C_ and *R*_2_ play an important role in the triggering voltage of the proposed B-TVS-D. An increase in *R*_1A_/*R*_1C_ or decrease in *R*_2_ can lead to decrease of the triggering voltage. In addition, the proposed B-TVS-D can be fabricated on the traditional p-GaN HEMT platform, making the ESD design of the GaN-based power system more convenient.

## Data Availability

Data available on request, having regard to restrictions, e.g., privacy or ethical.
